# ﻿*Phaeocerosperpusillus* var. *﻿scabrellus* (Notothyladaceae, Anthocerotophyta), a new taxon from northern Thailand

**DOI:** 10.3897/phytokeys.244.124080

**Published:** 2024-07-23

**Authors:** Orawanya Suwanmala, Juan Carlos Villarreal A., Fay-Wei Li, Sahut Chantanaorrapint

**Affiliations:** 1 PSU Herbarium, Division of Biological Science, Faculty of Science, Prince of Songkla University, Hat Yai, Songkhla 90110, Thailand Prince of Songkla University Hat Yai, Songkhla Thailand; 2 Département de Biologie, Pavillon C.-E. Marchand Université Laval, Québec, Canada Département de Biologie, Pavillon C.-E. Marchand Université Laval Québec Canada; 3 Boyce Thompson Institute, Ithaca, New York, USA Boyce Thompson Institute Ithaca, New York United States of America; 4 Plant Biology Section, Cornell University, Ithaca, New York, USA Cornell University Ithaca, New York United States of America

**Keywords:** Endangered, hornwort, low-copy nuclear markers, new variety, spore ornamentation

## Abstract

A new variety of hornwort from northern Thailand, Phaeocerosperpusillusvar.scabrellus is described based on morphological characters and molecular phylogenetic analyses. In this study, phylogenetic analyses supported that the new variety is closely related to P.perpusillusvar.perpusillus. Morphologically, it is distinguished from the autonimic variety in nearly smooth spores under light microscope. A taxonomic description, illustrations, and light and scanning electron micrographs are provided. In addition, the new variety is assessed as Endangered (EN), demonstrating its rarity by being currently known from only three subpopulations.

## ﻿Introduction

*Phaeoceros* Prosk. (Notothyladaceae) is the third largest genus of hornwort with about 34 currently accepted species worldwide (Söderström et al. 2016) and widely distributed in both the Northern and Southern Hemispheres (Cargill and Fuhrer 2008). The genus is defined by a smooth solid thallus, single chloroplast per cell, presence of a pyrenoid, antheridial chambers with usually (1–)2–6(–8) antheridia, irregularly arranged jacket cells of the antheridia, and yellow spores (Duff et al. 2007; Cargill and Fuhrer 2008; Chantanaorrapint 2009; Villarreal et al. 2010). In Thailand three species have been reported: *P.carolinianus* (Michx.) Prosk., *P.himalayensis* (Kashyap) Prosk. ex Bapna & G.G.Vyas, and *P.perpusillus* Chantanaorr. (Lai et al. 2008; Chantanaorrapint 2009; Chantanaorrapint et al. 2015).

During the bryological surveys in Chiang Mai Province, northern Thailand, some interesting specimens of the hornwort genus *Phaeoceros* were collected. These specimens resemble *P.perpusillus*, an endemic species of northern Thailand, in having small gametophytes, short sporophytes (usually less than 1 cm long), yellow spores, and subquadrate pseudoelater cells. Following a detailed comparison with closely related taxa, we here describe and illustrate these specimens as a new variety of *P.perpusillus*. We also used for the first time three hornwort specific low-copy nuclear markers. In theory, low-copy nuclear genes tend to have higher mutation rates than organellar genes, resulting in more variable sites that can be used for phylogenetic reconstruction, especially at species-level (Sang 2002; Feliner and Rosselló 2007). However, despite the advantages of biparental inheritance, which provides a more comprehensive view of the genetic history and evolution, low-copy nuclear genes have not been widely used (Zhang et al. 2012). In this study, we combined selected low-copy nuclear sequences with chloroplast sequences to enhance the resolution of our phylogenetic analyses. By using both chloroplast and nuclear markers, our study aims to explore alternative genetic regions for species-level phylogenies, thus providing a greater understanding of hornwort evolution.

## ﻿Materials and methods

### ﻿Morphological study

This study is based on recent collections from Thailand. Voucher specimens of the new species are deposited in BKF, NICH, and PSU herbaria. Morphological and anatomical characters were studied using stereo- and compound microscopes. The distinctive characters of the species were photographed using an Olympus BX51 microscope equipped with a DP74 digital camera and illustrated with the aid of an Olympus drawing tube. Mature spores were coated with a thin layer of gold and examined under a FEI Quanta 400 scanning electron microscope operating at 20 kV. The preliminary conservation status was assessed following the International Union for Conservation of Nature (IUCN) Red List criteria (IUCN 2022) and using GeoCAT (Bachman et al. 2011) to calculate the area of occupancy (AOO) and extent of occurrence (EOO). In addition, distribution and ecological data were compiled; descriptions and illustrations are provided.

### ﻿Taxon sampling

Twenty-seven samples of *Phaeoceros* spp. were included in our molecular dataset. Additionally, *Notothylaslevieri* Schiffn. ex Steph. and *Paraphymatoceros* sp. were employed as the outgroup. List of newly generated sequence used in the phylogeny with voucher information and GenBank accession numbers are provided in Table 1.

**Table 1.** List of newly generated sequence used in the phylogeny with voucher information and GenBank accession numbers.

Taxa	Collector	*rbc*L	L138	L178	L315
*Paraphymatoceros* sp. Mexico	*Morales 22*	**OR943578**	**PP481902**	**PP471573**	**PP471590**
*Phaeoceroscarolinianus* Thailand1	*Chantanaorrapint & Suwanmala 3955*	**OR943588**	**PP481909**	**PP471580**	**PP471598**
*P.carolinianus* Thailand2	*Chantanaorrapint & Suwanmala 3909*	**OR943586**	**PP481911**	**PP471581**	**PP471600**
*P.carolinianus* Thailand3	*Chantanaorrapint & Suwanmala 4057*	**OR943585**	**PP481913**	**PP471583**	**PP471602**
*P.carolinianus* India1	*Villarreal & Uniyal1314*	**OR943596**	**PP481901**	**PP471572**	**PP471589**
*P.carolinianus* India3	*Villarreal 1233*	**OR943593**	**PP481904**	**PP471575**	**PP471593**
*P.carolinianus* India2	*Duckett IE45*	**OR943592**	**PP481905**	**PP471576**	**PP471594**
*P.carolinianus* Czech Republic	*Kopal* s.n.	**OR943591**	**PP481906**	**PP471577**	**PP471595**
*P.carolinianus* Indonesia	*Gradstein 12362*	**OR943595**	-	-	**PP471591**
*P.carolinianus* Vietnam	*Suwanmala 849*	**OR943582**	**PP481916**	**PP471585**	**PP471604**
*P.exiguus* Thailand2	*Chantanaorrapint & Suwanmala 4129*	**OR943580**	**PP481918**	**PP471587**	**PP471606**
*P.himalayensis* India	*Duckett IW15*	**OR943594**	**PP481903**	**PP471574**	**PP471592**
*P.kashyapii* Thailand	*Chantanaorrapint & Suwanmala 3901*	**OR943589**	**PP481908**	**PP471579**	**PP471597**
*P.mohrii* USA	*Doyle 11341*	**OR943590**	**PP481907**	**PP471578**	**PP471596**
P.perpusillusvar.perpusillus Thailand2	*Chantanaorrapint & Suwanmala 3883*	**OR943587**	**PP481910**	-	**PP471599**
P.perpusillusvar.perpusillus Thailand3	*Chantanaorrapint & Suwanmala 4076*	**OR943584**	**PP481914**	**PP471584**	**PP471603**
P.perpusillusvar.scabrellus Thailand1	*Chantanaorrapint & Suwanmala 4077*	**OR943583**	**PP481915**	-	-
P.perpusillusvar.scabrellus Thailand2	*Chantanaorrapint & Suwanmala 4116*	**OR943581**	**PP481917**	**PP471586**	**PP471605**
*Phaeoceros* sp. Thailand	*Chantanaorrapint & Suwanmala 4488*	**OR943579**	**PP481919**	**PP471588**	**PP471607**

### ﻿DNA extraction, amplification, and sequencing

Total genomic DNA from silica gel-dried sporophytes was extracted using E.Z.N.A. Plant DNA kit (Omega Bio-Tek, USA) following manufacturer’s protocols. An alignment of more than 400 loci from a probe developed and explained by Breinholt et al. (2021) was used to reconstruct the phylogeny of all hornworts (Peñaloza-Bojacá et al. submitted). From the alignment we selected three loci found in *Phaeoceros* species (L138, L178, and L315) and designed internal primers using Geneious 2021.1.1. Amplification was accomplished using four primers listed in Table 2, one for the *rbc*L gene as described in Duff et al. (2004) and three primers newly designed for *Phaeoceros* nuclear loci (L138, L178 and L315). The conditions for PCR were as follows: (1) for *rbc*L, L138 and L315: initial denaturation for 3 min at 94 °C, followed by 35 cycles of 1 min at 94 °C, 30 s at 55 °C, 1 min at 72 °C, and final extension for 10 min at 72 °C, (2) for L178: initial denaturation for 3 min at 94 °C, followed by 35 cycles of 1 min at 94 °C, 30 s at 58 °C, 1 min at 72 °C, and final extension for 10 min at 72 °C. The final products were incubated at 10 °C to complete the reaction. The PCR products were purified and sequenced by Plate-forme d’analyses génomiques (Quebec, Canada), except for *O. Suwanmala 849*, *S. Chantanaorrapint & O. Suwanmala 4116, 4129, 4488* which were performed by the Macrogen sequencing service (Macrogen, Korea).

**Table 2.** Primer sequence used for PCR amplification and sequencing.

Region	Sequence 5’-3’	Reference
**L138**		
Phaeoceros_L138_58F	TTG TCC TGA ATT CAC GTG GT	This study
Phaeoceros_L138_607R	GCT TTG CTA GGG TCT GGT AAG A	This study
**L178**		
Phaeoceros_L178_232F	CTC GGG GAT GAG CGG GAC	This study
Phaeoceros_L178_1088R	GCT TCA AGA GAT GGC TCC TT	This study
**L315**		
Phaeoceros_L315_676F	GGA TTT TGG GGA CTT GCA CA	This study
Phaeoceros_L315_1325R	CTT CTG CCC AAC AAC AGG AG	This study
***rbc*L**		
rbcL2_16F	GAG ACT AAA GCA GGT GTT GGA	Duff et al. (2004)
rbcL_976R	ACA CGA AAG TGA ATA CCA TG	Duff et al. (2004)

Forward and reverse sequences were edited initially and assembled using Geneious 2021.1.1. We gathered published data from six samples generated by Breinholt et al. (2021), UFG_393201_P02_WH01, UFG_393201_P02_WA02, UFG_393201_P02_WB02, UFG_393201_P02_WH02, UFG_393201_P02_WG02, UFG_393201_P02_WD01, one sample of UFG_393202_P054_WD04 generated by Bechteler et al. (2023), and three samples, UFG_393202_P033_WD01, UFG_393202_P054_WE04, UFG_393202_P033_WC01, generated by Peñaloza-Bojacá et al. (submitted). Nineteen newly generated sequences (Table 1) and ten published sequences were aligned using the Geneious alignment algorithm with default settings. Uncertain alignment positions and columns displaying a large number of gaps were excluded from the phylogenetic assessments. Any incomplete sequence segments and nucleotide gaps were treated as missing data.

### ﻿Phylogenetic analysis

A maximum likelihood (ML) analysis was performed in RAxML HPC BlackBox v.8.2 (Stamatakis 2014) using GTR+I+GAMMA substitution model following default setting with 1000 bootstrap replications. The best model scheme of each partition was carried out in Partitionfinder 2 (Lanfear et al. 2016). Bayesian analysis was performed in MrBayes 3.2 (Ronquist et al. 2012) using Markov chain Monte Carlo (MCMC) searches with two runs and four chains of 3,500,000 generations. Trees were sampled every 1000^th^ generation and the first 10% of sampled trees were discarded as a burn-in to ensure a convergence of the analyses. We used Tracer 1.5 (Rambaut et al. 2018) to evaluate the burn-in and convergence. Figtree was used to graph and edit trees (Rambaut 2017). Both maximum likelihood and Bayesian analyses were performed on CIPRES Science Gateway (Miller et al. 2010).

## ﻿Results

**Figure 1.**
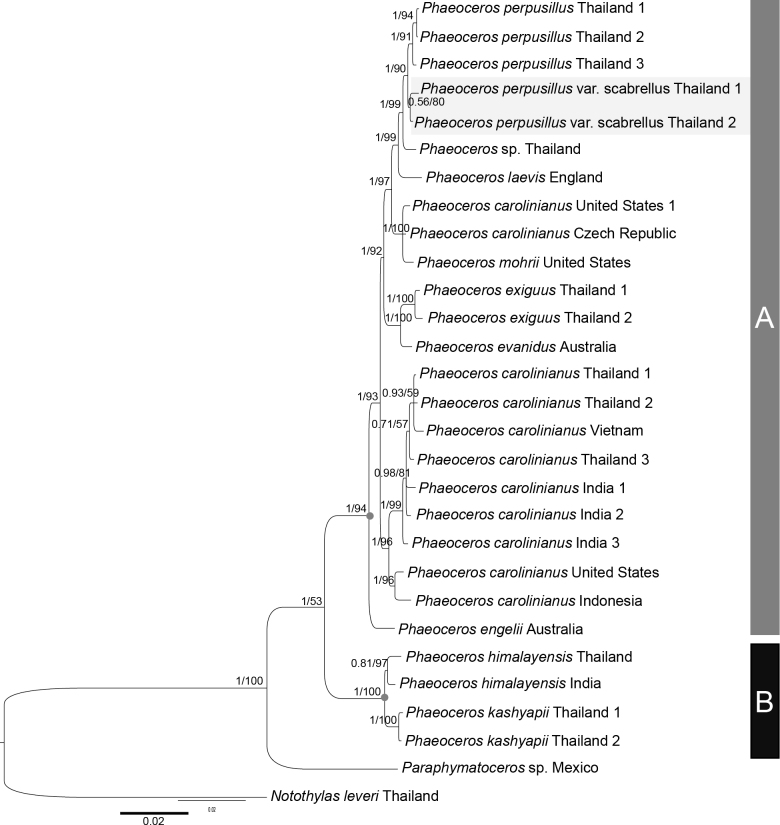
Majority rule consensus tree of phylogenetic relationships of *Phaeoceros* derived from Bayesian analyses of the combined dataset of *rbc*L, L138, L178, and L315 genes. Bayesian posterior probability values (PP) and bootstrap percentages values (MLBS) are shown on branches respectively. Nucleotide substitution rates indicated below the tree. Clade A include papillate spore *Phaeoceros* and the new variety (highlighted), and Clade B include non-papillate spore *Phaeoceros*.

A concatenated dataset of the coding region of one plastid and three nuclear markers (*rbc*L, L138, L178 and L315) contained 2856 characters (892, 549, 781, and 634 characters respectively). Tree topologies generated by Bayesian inference (**BI**) and maximum likelihood exhibited congruent patterns shown in Fig. 1, with posterior probabilities (**PP**) and maximum likelihood bootstrap values (**MLBS**) plotted on the branches. The monophyly of the genus *Phaeoceros* is well supported by posterior possibility (PP = 1) but weakly supported by maximum likelihood analysis (MLBS = 53). In the tree topology (Fig. 1), *Phaeoceros* was divided into two major lineages with strong support, clade A including twenty-three terminals, containing the new taxon and other papillate spore *Phaeoceros* (PP = 1, MLBS = 94), and clade B comprising four terminals of non-papillate spore *Phaeoceros* including *P.himalayensis* and *P.kashyapii* A.K. Asthana & S.C. Srivast. (PP = 1, MLBS = 100).

The inclusion of P.perpusillusvar.scabrellus and its autonimic variety in the data matrix resolves this species lineage as monophyletic with good support (PP = 1, MLBS = 90). The new variety is recovered as sister to the autonimic variety with less posterior probability and bootstrap support (PP = 0.56, MLBS = 80).

### ﻿Taxonomic treatment

#### ﻿ Phaeoceros perpusillus Chantanaorr. var. scabrellus

Taxon classificationPlantaeNotothyladalesNotothyladaceae

Suwanmala & Chantanaorr. var. nov.

F7B035B5-CAA1-50E9-B59B-166914AA2CF5

Figs 2 , 3 , 4

##### Type.

Thailand. Chiang Mai Province: Doi Suthep-Pui, Bhu Bing Palace, 1400 m, 18 October 2020, *S. Chantanaorrapint & O. Suwanmala 4077* (holotype: PSU!; isotype: BKF!, NICH!).

**Figure 2.**
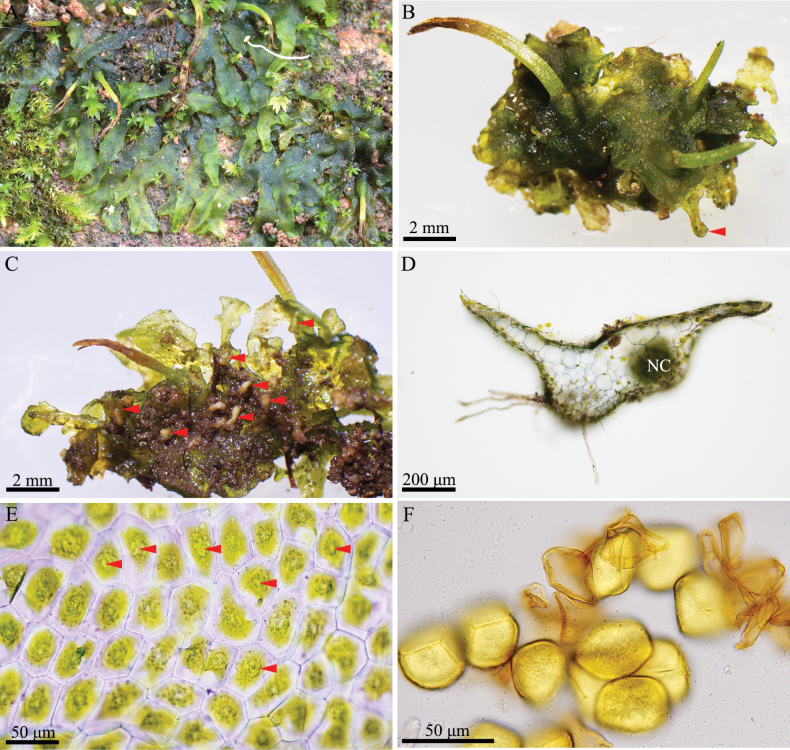
Phaeocerosperpusillusvar.scabrellus**A** plants in natural habitat **B** dorsal view of thallus showing marginal tubers (arrow) **C** ventral view of thallus showing ventral tubers (arrows) **D** cross-section of thallus showing the large dark lump of Nostoc colony (NC = Nostoc colony) **E** dorsal epidermal cells of thallus showing a single chloroplast with pyrenoid (arrows) per cell **F** spores and pseudoelaters. Photos by O. Suwanmala (**A** from *S. Chantanaorrapint & O. Suwanmala 4116***B–F** from *S. Chantanaorrapint & O. Suwanmala 4077*).

##### Diagnostic.

Phaeocerosperpusillusvar.scabrellus is similar to the autonimic variety but differs in nearly smooth spores under light microscope (or vermiculate under SEM), whereas the autonimic variety have pluripapillae on the distal surface and vermiculate on the proximal.

**Figure 3.**
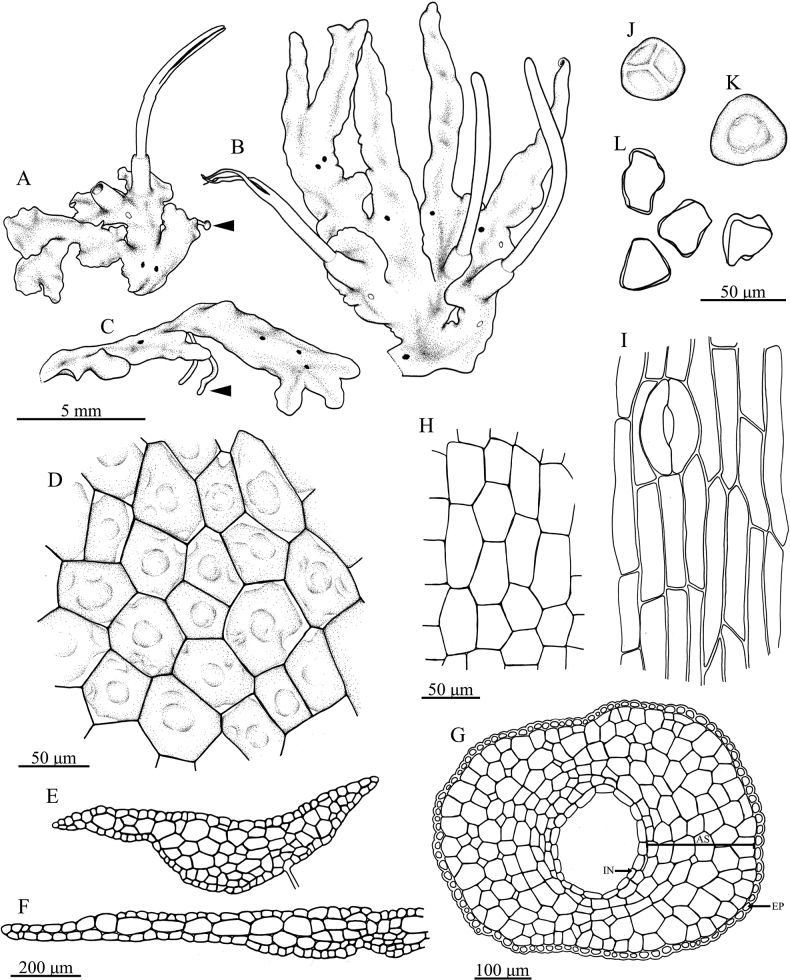
Phaeocerosperpusillusvar.scabrellus**A** gametophyte forming half-rosettes with sporophyte (arrow indicate tuber) **B** ensiform thalli and sporophytes **C** gametophyte showing ventral tuber (arrow) **D** dorsal epidermal cells of thallus **E, F** cross sections of thalli **G** cross section of sporangium (AS = assimilative tissue, EP = epidermal cell of capsule, IN = inner most sporangium wall) **H** inner most cells of sporangium wall **I** epidermal cells of capsule with stoma **J** proximal view of spore **K** distal view of spore **L** pseudoelaters. All from holotype and drawings by O. Suwanmala. (All drawing from *S. Chantanaorrapint & O. Suwanmala 4116*).

##### Description.

***Thallus*** yellowish-green to dark green in fresh material, dull green to blackish- brown in dry material, prostrate or moderately adhering to the substratum, solid, ecostate, orbicular to sub-orbicular, dichotomously branched into several lobes, with a smooth dorsal surface; lobes ensiform or sometimes fan-shaped, up to 0.8 mm long, 1–3 mm wide; margins wavy, nearly entire to shallowly crenulate; apex flat, rarely ascending, occasionally tapering into apical tubers; tubers sometimes present on ventral surface. Thallus in cross section plano-convex to concave-convex, 4–10 cells thick in the middle region, without mucilage cavities. Dorsal epidermal cells rectangular to heptagonal, 28–75 × 25–50 µm, thin-walled, smooth. Chloroplast one per cell, large, occupying almost entire cell, variable in shape; pyrenoid present. ***Nostoc colonies*** scattered through the ventral side of thallus, appearing as dark dots. ***Rhizoids*** hyaline or pale brown along ventral surface, inner wall smooth or tuberculate. ***Sexuality*** monoicous. Androecia scattered and slightly raised over the dorsal surface of thallus, 2–3 antheridia per chamber; antheridia subglobose to globose, exposed at maturity, irregularly arranged jacket cells, shortly stalked, stalk with quadriseriate cells. Archegonia embedded in thallus, connected to the upper surface, scattered near the lobe of thallus. ***Involucre*** solitary, conical-cylindrical, up to 2 mm long, 2–4 cells thick, mouth smooth to crenulate. ***Sporophytes*** capsule somewhat inclined, stout to narrowly cylindrical, 0.5–1(–1.2) cm long, yellow at apex, dehiscing from top toward base, bivalves rarely twisted when dry; epidermal cells of capsule elongate-rectangular, 68–200 × 12–30 µm, thick-walled, stomata present with two reniform guard cells, surrounded by 5–8 epidermal cells; assimilative layers 3–6 cells thick in cross section; the innermost capsule cells dark brown, subquadrate to rectangular; 27–67 × 22–53 µm; columella well-developed, red-brown, consisting of 16 cells (4 × 4 lines of cells) in cross section. ***Spores*** unicellular, yellow, rounded-triangular in polar view, equatorial diameter 32–50 µm in diameter, nearly smooth under light microscope (LM), proximal surface with a distinct trilete mark, bordered by vermiculate strip on each side of trilete mark, each facet covered with fine vermiculate pattern; distal surface with a slightly dome-like region at the center, more densely vermiculate than proximal surface, sometimes with minute granules. ***Pseudoelaters*** light brown or yellowish-brown at maturity, thin-walled, occasionally branched; pseudoelater cells subquadrate to short rectangular, 30–45 × 25–30 μm, without helicoidal band.

**Figure 4.**
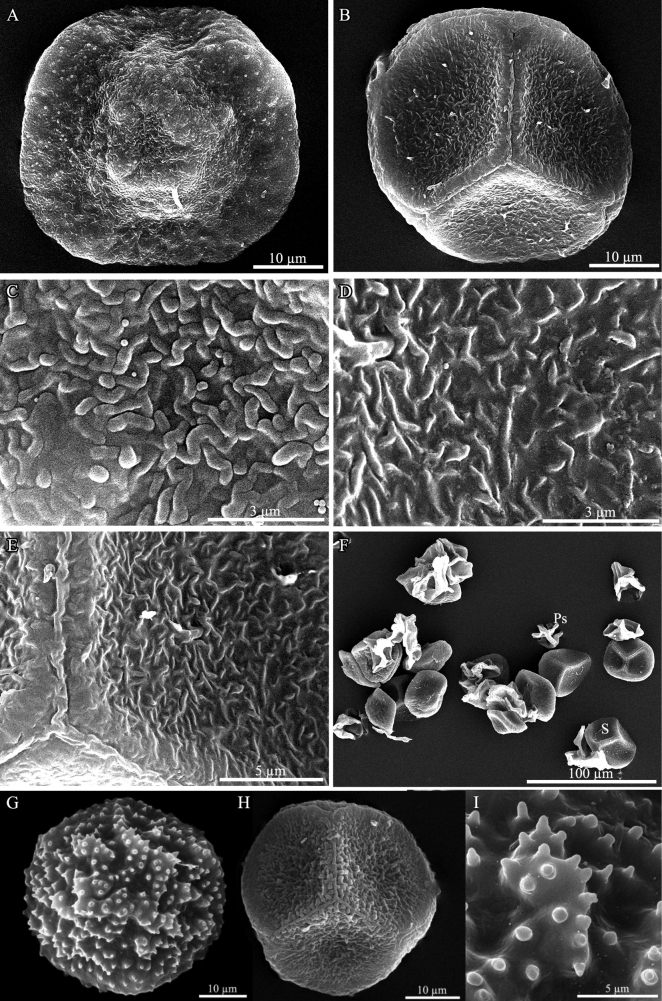
Scanning electron micrographs of spores **A–F**Phaeocerosperpusillusvar.scabrellus**A** distal view of spore showing a central hump-like projection **B** proximal view of spore with a distinct triradiate mark **C** close-up of distal surface showing packed vermiculae **D** close-up of proximal surface showing loosely arranged vermiculae **E** proximal surface showing trilete mark and loosely arranged vermiculae **F** spores and pseudoelaters **G–I**P.perpusillusvar.perpusillus**G** distal view of spore **H** proximal view of spore **I** close-up of distal surface showing pluripapillae. Photos by O. Suwanmala. (**A–F** images from *S. Chantanaorrapint & C. Promma 3129***G–I** images from *S. Chantanaorrapint & O. Suwanmala 3883*).

##### Etymology.

The epithet of the variety refers to scabrate ornamentation observed under light microscope.

##### Habitat and distribution.

Phaeocerosperpusillusvar.scabrellus is currently known only from northern Thailand. It grows on disturbed soil and sandstone in open site in grassland, pine-oak mixed montane deciduous forests at elevation of 1390–2100 m. It may grow associated with other bryophytes such as *Anthocerossubtilis* Steph., *Notothylaslevieri*, *N.orbicularis* (Schwein.) Sull. ex A.Gray, and *P.carolinianus*.

##### Conservation status.

This variety is currently known from three subpopulations, which are in protected areas (Chiang Dao Wildlife Sanctuary and Doi Suthep-Pui National Park). One of the subpopulations is located in a camping area, which is a common visiting site for tourists and dominated by *Ageratinaadenophora* (Spreng.) R.M.King & H.Rob. (invasive species). Therefore, habitat quality is threatened by trampling and other destructive activities potentially caused by regular visits by tourists to the area, and invasive plant species. Together, these have the potential to cause a population reduction. The other subpopulation is also somewhat disturbed by human activities such as shifting cultivation. The extent of occurrence (EOO) of P.perpusillusvar.scabrellus is estimated to be 262.925 km^2^ and its area of occupancy (AOO) is estimated to be 12 km^2^, which falls within the limits for Endangered status under criterion B1 and B2 of IUCN Red List Categories and Criteria. Conservation efforts should focus on implementing strict regulations to reduce the impact of human activity and controlling invasive species, while also raising awareness among local communities about the importance of protecting the habitat.

##### Additional specimens examined.

**Thailand**. Chiang Mai Province: Chiang Dao Wildlife Sanctuary, 1700–2000 m, 1 November 2013, *S. Chantanaorrapint & C. Promma 3125B, 3129, 3216* (PSU); Doi Suthep-Pui National Park, Doi Mon Long Viewpoint, 1390 m, 4 November 2015, *S. Chantanaorrapint & W. Juengprayoon 143B* (PSU); 15 November 2020, *S. Chantanaorrapint & O. Suwanmala 4089, 4090* (PSU); Bhu Ping Palace, 1400 m, 8 September 2013, *S. Rattanamanee 3* (PSU); 18 October 2020, *S. Chantanaorrapint & O. Suwanmala 4077* (PSU); 5 October 2021, *S. Chantanaorrapint & O. Suwanmala 4116* (PSU).

## ﻿Discussions

Phaeocerosperpusillusvar.scabrellus is morphologically similar to the autonimic variety which is endemic to northern Thailand (Chantanaorrapint 2009). These two varieties share some common features, viz. small orbicular gametophytes (Fig. 2A, B), monoicous sexual condition, very short capsules (usually less than 1 cm long) (Figs 2B, 3A, B), yellow spores, and pseudoelater cells being subquadrate to short rectangular (Figs 2F, 3L). The new variety also resembles *P.exiguus* (Steph.) J. Haseg., a species found in Indonesia, New Caledonia and Taiwan (Hasegawa 1986, 1993; Siagian et al. 2021). They are monoicous, and have a small thallus, very short capsules, and small pseudoelaters. However, they can be distinguished by the spore ornamentation. Phaeocerosperpusillusvar.scabrellus is distinct from the autonimic variety and *P.exiguus* in nearly smooth spores under light microscope or vermiculate spores under SEM (Fig. 4A–F). In contrast, spores of P.perpusillusvar.perpusillus are pluripapillose on distal face and finely vermiculate on proximal face (Fig. 4G–I), while *P.exiguus* have button-like papillae on distal face and minutely papillae on proximal face.

In addition, the small plants of *P.carolinianus*, a common species, can be confused with *P.perpusillus* or *P.exiguus* in general appearance. The comparisons of morphological characters between these three monoicous species are summarized in Table 3.

**Table 3.** The comparisons of characters between P.perpusillusvar.scabrellus, P.perpusillusvar.perpusillus, *P.exiguus* and *P.carolinianus*.

Characters	P.perpusillusvar.scabrellus	P.perpusillusvar.perpusillus	*P.exiguus*	*P.carolinianus*
Thallus	4–10 cells thick in the middle	6–9 cells thick in the middle	6–7 cells thick in the middle	8–13 cells thick in the middle
Capsule placement	oblique	oblique	usually erect	Erect
Capsule length	usually less than 1 cm	less than 1 cm	up to 1.5 cm	usually more than 1.5 cm
Involucre	up to 2 mm high	1–2 mm high	1–2 mm high	2–4 mm high
Spore diameter	32–50 µm	40–47 µm	40–42 µm	30–37 µm
Distal surface of spore	densely vermiculate, with minute granules	pluripapillose	dense clusters of button-like papillae	densely spinose
Proximal surface of spore	loosely vermiculate in each facet	finely vermiculate in each facet	minutely papillate throughout each facet	minutely papillate in central part of each facet
Pseudoelaters (length/width ratio)	1–1.5 ×	1.5–2.5 ×	1.2–2 ×	≥4 ×

Although both varieties of *P.perpusillus* have been reported only from the northern part of Thailand, P.perpusillusvar.perpusillus seems to have a wider range of distribution and is more abundant than the new variety. The new variety has been found in only three subpopulations, overlapping with the autonimic variety, which is assessed as Endangered (EN) according to IUCN Red List.

The placement of the new variety falls into the papillate spore *Phaeoceros* lineage (Fig. 1, clade A), despite the absence of spines or papillae on its spore surface which sets the new variety apart from other taxa. Within an assemblage of autonimic variety *P.perpusillus* and the new variety clade, the two taxa share a sister relationship with low support, and they show only one morphological difference in spore morphology. The vermiculate spore ornamentation observed in P.perpusillusvar.scabrellus seems to be an unusual form of the autonimic variety. However, based on careful investigation, it becomes evident that the absence of papillae on the spore surface is consistently observed throughout the entire capsule and reveals a uniform pattern in each population. Spore morphology serves as a key trait to differentiate hornwort species, allowing two distinct spore ornamentations to be considered as separate taxa.

This proposal for the new variety’s classification was made due to its gametophyte and sporophyte morphological similarity to the autonimic variety with the exception of the spore ornamentation, and was also supported by phylogenetic inference, and the shared distribution area.

## Supplementary Material

XML Treatment for Phaeoceros perpusillus Chantanaorr. var. scabrellus

## ﻿Additional information

### Author ORCIDs

Orawanya Suwanmala https://orcid.org/0000-0002-1113-7614

Juan Carlos Villarreal A. https://orcid.org/0000-0002-0770-1446

Fay-Wei Li https://orcid.org/0000-0002-0076-0152

Sahut Chantanaorrapint https://orcid.org/0000-0002-9739-0994

### Data availability

All of the data that support the findings of this study are available in the main text.
